# Complexity of the Yellowstone Park Volcanic Field Seismicity in Terms of Tsallis Entropy

**DOI:** 10.3390/e20100721

**Published:** 2018-09-20

**Authors:** Kalliopi Chochlaki, Georgios Michas, Filippos Vallianatos

**Affiliations:** UNESCO Chair on Solid Earth Physics & Geohazards Risk Reduction, Technological Educational Institute of Crete, Crete 73100, Greece

**Keywords:** Tsallis entropy, Non Extensive Statistical Physics, Yellowstone park, volcano seismicity, earthquake swarms

## Abstract

The Yellowstone Park volcanic field is one of the most active volcanic systems in the world, presenting intense seismic activity that is characterized by several earthquake swarms over the last decades. In the present work, we focused on the spatiotemporal properties of the recent earthquake swarms that occurred on December–January 2008–2009 and the 2010 Madison Plateau swarm, using the approach of Non Extensive Statistical Physics (NESP). Our approach is based on Tsallis entropy, and is used in order to describe the behavior of complex systems where fracturing and strong correlations exist, such as in tectonic and volcanic environments. This framework is based on the maximization of the non-additive Tsallis entropy *S_q_*, introducing the *q*-exponential function and the entropic parameter *q* that expresses the degree of non-extentivity of the system. The estimation of the *q*-parameters could be used as a correlation degree among the events in the spatiotemporal evolution of seismicity. Using the seismic data provided by University of Utah Seismological Stations (UUSS), we analyzed the inter-event time (*T*) and distance (*r*) distribution of successive earthquakes that occurred during the two swarms, fitting the observed data with the *q*-exponential function, resulting in the estimation of the Tsallis entropic parameters *q_T_*, *q_r_* for the inter-event time and distance distributions, respectively. Furthermore, we studied the magnitude-frequency distribution of the released earthquake energies *E* as formulated in the frame of NESP, which results in the estimation of the *q_E_* parameter. Our analysis provides the triplet (*q_E_*, *q_T_*, *q_r_*) that describes the magnitude-frequency distribution and the spatiotemporal scaling properties of each of the studied earthquake swarms. In addition, the spatial variability of *q_E_* throughout the Yellowstone park volcanic area is presented and correlated with the existence of the regional hydrothermal features.

## 1. Introduction

**Figure 1:**
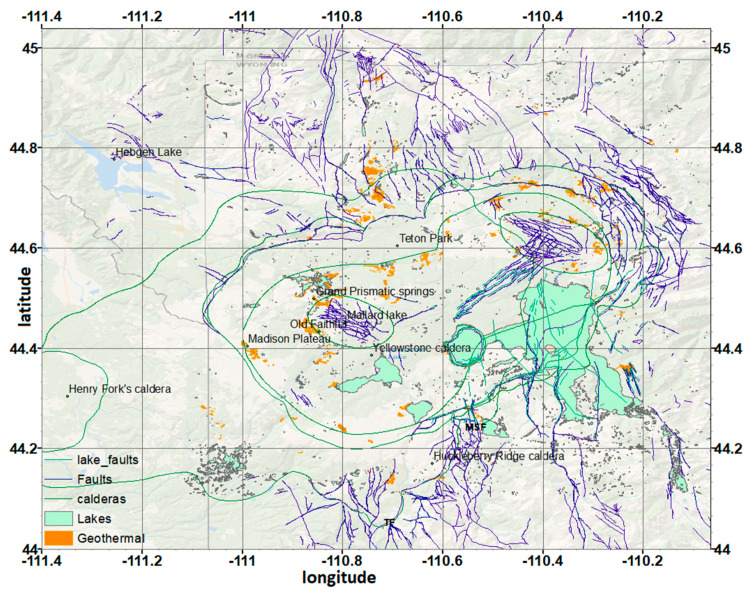
Map with the faults and geothermal areas of the Yellowstone volcanic field.

The Yellowstone national park is one of the USA most visited parks and a UNESCO world heritage site. It exceeds approximately 8987 km^2^ and its biggest part is located in the northwest corner of Wyoming, but portions extend into the states of Idaho and Montana. Apart from the wildlife and the physical beauty of the area, the park is widely known due to the vast number of geysers, hot springs, and other geothermal features (Figure 1). The Yellowstone park volcanic field presents intense seismic activity and it is categorized as a “supervolcano”, representing one of the largest silicic volcanic systems in the world [1,2].

Three cataclysmic caldera-forming eruptions determined the evolution of the field in the past two million years. The first ash-flow sheet of the Yellowstone Group, the Huckleberry Ridge Tuff, erupted 2.1 million years ago. This extraordinary eruption buried an area of 15,500 km^2^ in a period of time so short that no erosion or cooling of earlier parts of the deposit occurred before completion of the eruption, i.e., certainly within a few hours or days [2]. As a result, the magma chamber roof collapsed, forming the first cycle caldera of the plateau. During the second cycle, the main volcanic activity was noticed west of the plateau at the Island Park area, where the eruption of the Messa Falls Tuff that occurred 1.3 million years ago formed the “Henry Fork” caldera, with a diameter of 16 km [2,3]. The Mesa Falls Tuff, although having less volume and covering less area than the Huckleberry Ridge, is a major ash-flow sheet, and its eruptive volume was greater than 280 km^3^. The most recent eruption, 0.64 million years ago, formed the Lava Creek Tuff and the Yellowstone caldera, which is the central feature of the field [4] (Figure 1). Several precaldera rhyolitic lava flows are known to have occurred in the field, which in addition to the existence of a growing ring-fracture system, indicate continuous magmatic intrusions that led to the expansion of the magma chamber. Subsequently, its catastrophic eruption produced the Lava Creek Tuff that later collapsed, resulting in the formation of the Yellowstone caldera.

**Figure 2:**
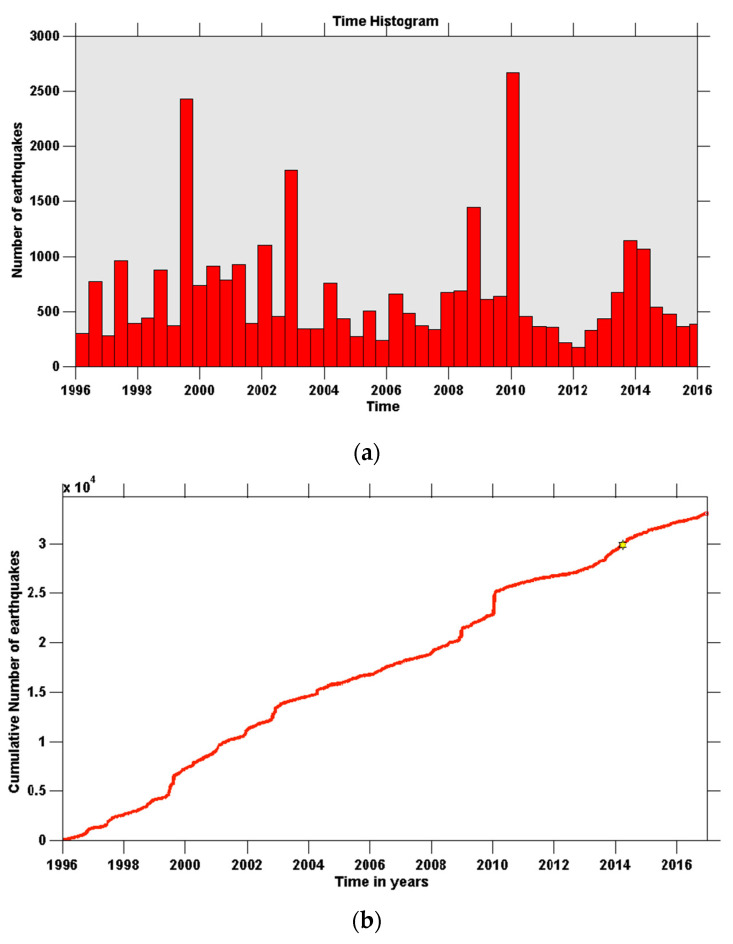
Seismicity rate (**a**) and Cumulative number of earthquakes (**b**) in the Yellowstone volcanic field from 1996 to 2016, for all the events with magnitude M > M_c_ = 1.5 (see text and [33]).

The Yellowstone Plateau is one of the most seismically-active areas of the western U.S., and is part of the distinct N–S band of intraplate seismicity known as the Intermountain seismic belt [5]. Earthquakes in Yellowstone generally have small magnitudes, but a few strong events have occurred over the years. A characteristic example of a strong earthquake that affected the whole region in the vicinity is the Hebgen Lake earthquake with magnitude M 7.5 that occurred in August 1959, just northwest of the boundary of the Yellowstone National Park, which resulted in a rich aftershock sequence within the active seismic zone [6]. Smaller magnitude M earthquakes are widely-observed in the neighboring faults, as are many intracaldera shallow earthquakes with earthquake magnitude, M ≤ 3 [7,8]. In Figure 2, the Seismicity rate and the Cumulative number of earthquakes in the Yellowstone volcanic field from 1996 to 2016, are presented.

Another usual phenomenon in volcanic and geothermal areas, such as the Yellowstone, is the occurrence of earthquake swarms, i.e., numerous earthquakes that are generally shallow and are strongly clustered in both space and time. Commonly, these swarms are aligned on tectonic trends, though not necessarily on recognized faults, and are generally considered to be related to the migration of hydrothermal fluids or magma in the shallow crust [9].

The objectives of the present work are to study, in terms of Tsallis entropy, the spatial properties of magnitude–frequency distribution of seismicity in complex systems where fracturing and strong correlations exist, such as in the volcanotectonic Yellowstone system, along with the spatiotemporal properties of the swarms which occurred on the Yellowstone Lake during December–January 2008–2009 and the 2010 Madison Plateau using the ideas of Non-Extensive Statistical Physics (NESP) and Tsallis entropy [10,11,12], which is one of the entropies used within the family of generalized entropies (see [13,14,15,16,17]). Since the complexity issues related with Geophysical problems are not high-order issues, the applicability of Tsallis entropy in Earth physics has been demonstrated in a series of recent publications on seismicity [18,19,20,21,22], natural hazards [23,24], plate tectonics [25], geomagnetic reversals [26], volcanic systems [27], rock physics [28], applied geophysics [29], and fault-length distributions [30,31]. Using the seismic data provided by the University of Utah Seismological Stations (UUSS), we analyzed the inter-event time and distance distributions of the earthquakes that occurred during the two swarms, fitting the observed data with the *q*-exponential function that maximizes the Tsallis entropy, along with the magnitude-frequency distribution, as formulated in the frame of NESP [32]. Our analysis provides the triplet (*q_E_*, *q_T_*, *q_r_*) which describes each of the studied swarms, along with the spatial variability of *q_E_* throughout the Yellowstone volcanic area, which can be associated with the existence of the regional hydrothermal features.

## 2. Recent Earthquake Swarms in the Yellowstone Caldera

Earthquake swarms are defined as a sequence of events which are strongly clustered in time and space without a dominant large event, and with differences in magnitude that do not exceed 0.5 magnitude units between the largest and the second largest event [34]. According to their spatiotemporal properties, volcanic earthquake swarms can be divided in two main categories. The first includes the earthquakes occurring near the crater in shallow depths before the eruption of andesitic and dacitic volcanoes, where andesites, dacites and basalts define the main chemical composition of lavas, and are further used as characteristics for the classification of volcanoes [35]. Events of this category usually consist of earthquakes with maximum magnitudes of M 5–6, and whose sequences last from 1 to 5 weeks. The energy and frequency of the earthquakes are continuously increasing and reach their peak point at the eruption state. In the second category, the swarm’s events usually take place in basaltic and andesitic volcanoes before flank eruptions. The epicenters of the earthquakes are located 3 to 10 km from the point where the new crater is formed, the maximum magnitude M can be as high as 5–7, and the duration of the swarm varies from 1 to 10 days. Contrary to the behavior of the swarms that belong in the first category, in the second category the frequency and the maximum energy of earthquakes follow an increasing trend, reaching a peak point, which is followed by a gradual decrease, while a few hours before the flank fissure opens, their occurrence rate drops dramatically [35]. Generally, these swarms occur due to tectonic trends, not necessarily on faults, and are related to the migration of hydrothermal fluids or magma in the shallow crust.

**Figure 3:**
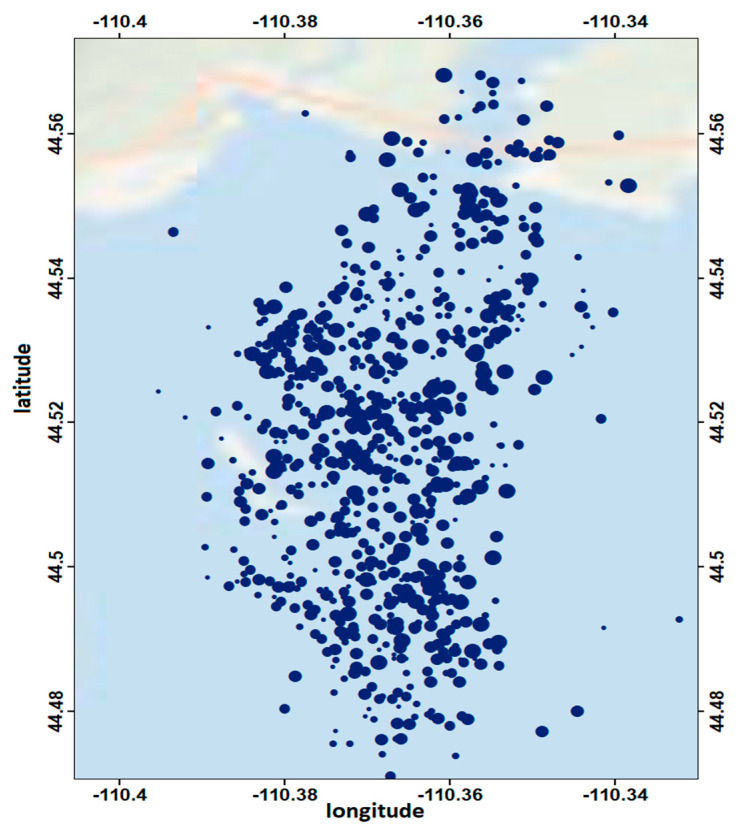
Seismicity map of the Yellowstone Lake swarm (2008–2009).

Here we focused on the analysis of two recent earthquake swarms, the Yellowstone Lake swarm (2008–2009) and the Madison Plateau swarm (2010). The first one initiated on 27 December 2008 and ended on 7 January 2009. It consisted of approximately 811 earthquakes, of which almost 20 events were felt in the Yellowstone National Park. Starting from the Lake area, seismicity migrated northward due to the possible involvement and migration of magmatic fluids [36] (Figure 3).

**Figure 4:**
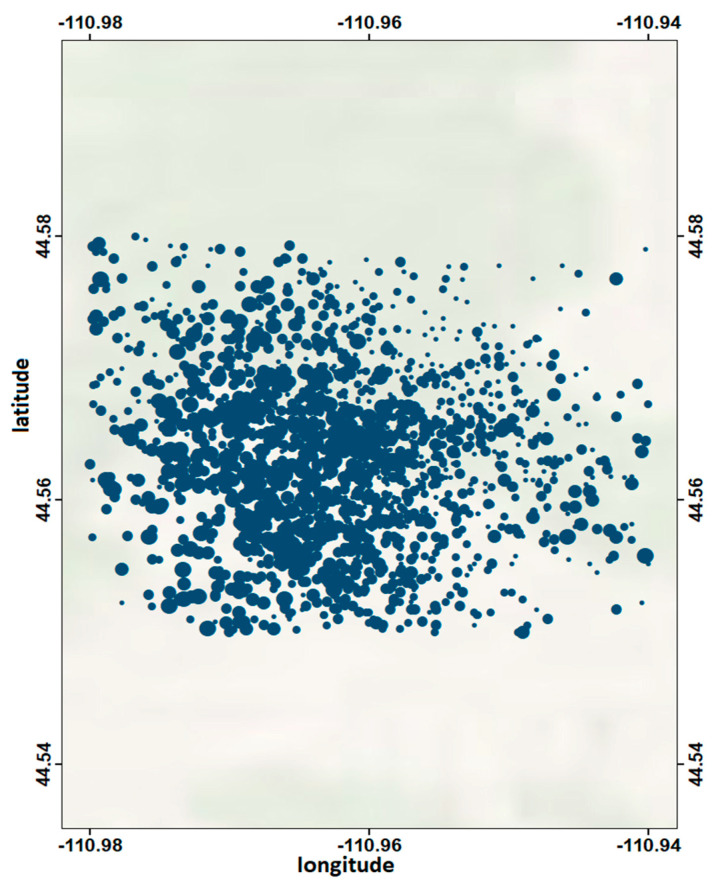
Seismicity map of the Madison Plateau earthquake swarm (2010).

The Madison Plateau swarm took place near the Northwest boundary of the Yellowstone caldera. It lasted almost 3 weeks, initiating on 17 January 2010; during this period, approximately 2250 earthquakes were recorded. The swarm started at about 10 km depth and expanded over time along a NNW striking plane [37] (Figure 4).

## 3. Yellowstone Seismicity in Terms of Tsallis Entropy

### 3.1. Spatiotemporal Scaling Properties of Yellowstone Swarms

In order to investigate the spatiotemporal scaling properties of the two earthquake swarms, a methodology based on Tsallis Entropy, which takes into consideration the complexity and the long-range interactions that exist among the earthquake sequences, was used. This framework is known as Non Extensive Statistical Physics (NESP), introduced by Tsallis in 1988 [10,11,12], and its cornerstone is the Tsallis entropy (*S_q_*), defined as: $S_{q}=k_{B}\frac{1-\sum_{i=1}^{W}p_{i}^{q}}{q-1}$, where *k_B_* is Boltzmann’s constant, *p_i_* is the set of probabilities, *W* is the total number of microscopic configurations, and *q* is the entropic index that expresses the degree of non-extensivity of the system [10,11]. For two probabilistic independent events *A* and *B*, the total entropy *S_q_* of the system *A* + *B* satisfies:

$$\frac{S_{q}\left(A+B\right)}{k_{B}}=\frac{S_{q}\left(A\right)}{k_{B}}+\frac{S_{q}\left(B\right)}{k_{B}}+\left(1-q\right)\frac{S_{q}\left(A\right)}{k_{B}}\frac{S_{q}\left(B\right)}{k_{B}}$$

The origin of non-addittivity comes from the last term on the right hand side of the above equation and is the fundamental principle of NESP [10]. When *q* = 1, the classic Boltzmann-Gibbs entropy is recovered, whereas the cases *q* > 1 and *q* < 1 correspond to sub-addittive and super-addittive systems respectively. For the probability distribution of a continuous variable *X* that can take any real value, the non-additive entropy *S_q_* is expressed by the integral formulation [10,11,12]: $S_{q}=k_{B}\frac{1-\int_{0}^{\infty }P^{q}\left(X\right)dX}{q-1}$, where *q* represents the entropic index.

Using the Lagrange multipliers technique, following [32,34], the physical probability is obtained as: $p\left(X\right)=\frac{\left[1-\left(1-q\right)\beta_{q}X\right]}{Z_{q}}=\frac{\exp_{q}\left(-\beta_{q}X\right)}{Z_{q}}$, where $Z_{q}$ refers to the *q*-partition function defined as: $\;Z_{q}=\int_{0}^{X_{max}}exp_{q}\left(-\beta_{q}X\right)dX\;$, with $\beta_{q}=\frac{\beta }{C_{q}+\left(1-q\right)\beta X_{q}}$ and $C_{q}=\int_{0}^{X_{max}}p^{q}\left(X\right)dX$, while the *q*-exponential function is defined [10,11] as:

expq(X)=[1+(1−q)X]1(1−q)       for  1+(1−q)X≥0expq(X)=0                        for  1+(1−q)X<0,

whose inverse is the *q*-logarithmic function: $ln_{q}\left(X\right)=\frac{1}{1-q}\left(X^{1-q}-1\right)$.

In the limit of *q*→1, the *q*-exponential and *q*-logarithmic functions lead to the ordinary exponential and logarithmic behavior, respectively. If *q* > 1, the *q*-exponential function presents power-law behavior with slope −1/(*q* − 1), whereas for *q* < 1 a cut-off appears. Since in the Earth sciences it is common to use cumulative distributions, in [38,39] it was shown that the cumulative distributions of inter-event distances *P*(>*r*) and times *P*(>*T*) between successive earthquakes are well described by the *q*-exponential distribution

(1) $$P\left(>X\right)=exp_{q}\left(-\frac{X}{X_{0}}\right)$$

The variable *X* refers to the inter-event times (*T*) or distances (*r*), while the *q*-value describes the spatiotemporal evolution and the degree of correlations, with *q_T_* > 1, for the inter-event times, and *q_r_* < 1 for the inter-event distances, respectively. Here, we use the cumulative distributions rather than the probability densities of the observed variables, as the scaling properties become more apparent due to the fact that the cumulative distributions produce smoother trends in the observed distribution. In addition, in [40] it was shown that both the cumulative distribution and the probability density of the earthquake inter-event times and magnitudes are well described by *q*-exponential functions derived in the frame of NESP, with similar *q*-values between the two, which describe the power-law asymptotic behavior of the observed distributions. 

The *q*-value is a quantitative measure of the scale of the interactions. A *q* value close to 1 indicates short-ranged correlations. As *q* increases, the physical state (in the sense of statistical physics) becomes much more unstable. The physical meaning underlying the non-extensive entropy formalism is that the final physical state can be considered as a collection of sub-states which have the sum of individual entropies larger than the entropy of the initial states [11]. The latter is straightforward from the concept of not additivity. Furthermore, we note that the entropic index *q* introduces a bias in the probabilities. Given the fact that generically 0 < *p_i_* < 1, we can conclude that $p_{i}^{q}>p_{i}$, if *q* < 1 and $p_{i}^{q}<p_{i}$, if *q* > 1. Therefore, *q* < 1 enhances the rare events, i.e., those which have probabilities close to zero, whereas *q* > 1 enhances the frequent events, i.e., those whose probabilities are close to unity.

**Figure 5:**
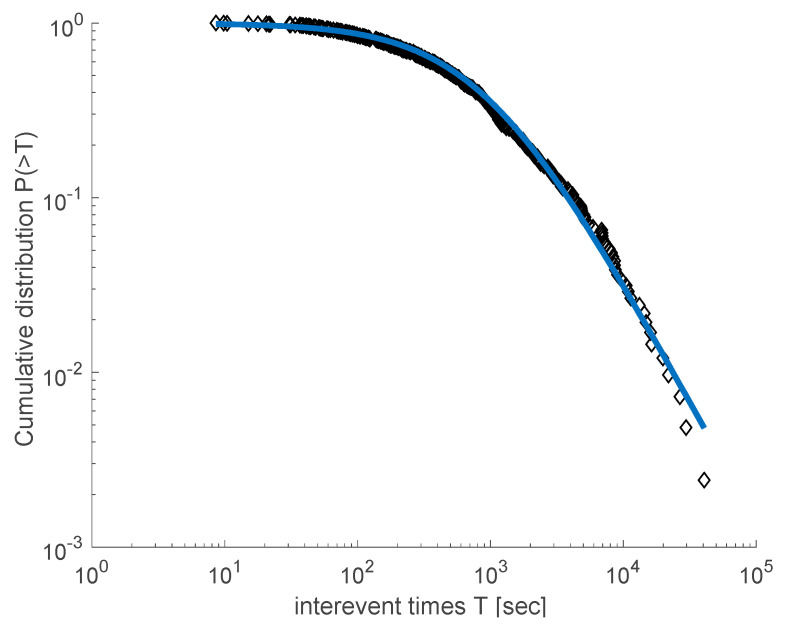
The cumulative distribution function of the inter-event times *T* for the 2008–2009 Yellowstone Lake swarm. The solid line is the *q*-exponential fitting for the values of *q_T_* = 1.715 and *T*_0_ = 653 s.

**Figure 6:**
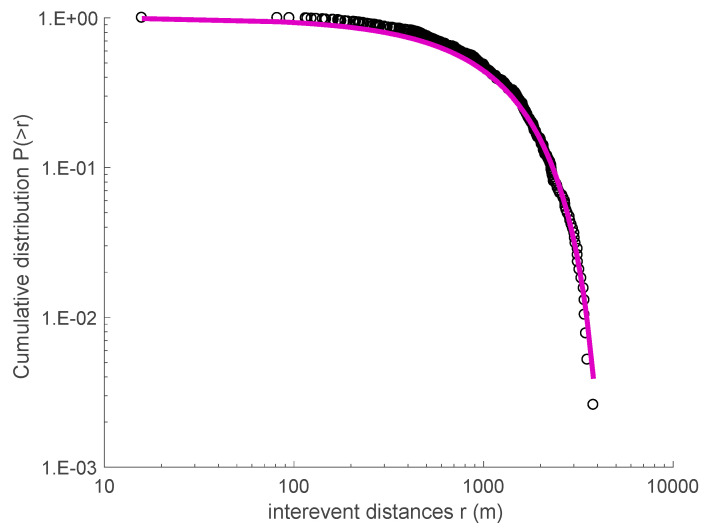
The cumulative distribution function for the inter-event distances *r* of the 2008–2009 Yellowstone Lake swarm. The solid line is the *q*-exponential fitting with *q_r_* equal to 0.710.

This approach has been applied in several studies that investigate the spatiotemporal properties of seismicity in a variety of tectonic environments and scales, and particularly from the laboratory to the regional and global scale [12,20,22,40,41,42]. We proceeded to analyze the temporal and spatial distributions of the 2008–2009 Yellowstone Lake swarm and the 2010 Madison Plateau swarm, using the data provided by the University of Utah Seismological Stations (UUSS). We note that since the beginning of 1995, the Yellowstone region catalog is estimated to be systematically complete above magnitude M_c_ = 1.5 [33]. For this reason, we used earthquakes with magnitude greater than M_c_ = 1.5 in order to proceed with the analysis of the catalog. Figure 5 presents the cumulative distribution *P*(>*T*) of inter-event times *T* for the Yellowstone Lake earthquake swarm (2008–2009). A *q*-exponential fitting using Equation (1) describes the observed data quite well, leading to *q_T_* = 1.715 ± 0.02 and *T*_0_ = (653 ± 5.2) s, while the inter-event distances distribution *P*(>*r*) is described quite well by the *q*-exponential function for *q_r_* = 0.71 ± 0.04 and *r*_0_ = (1610 ± 35) m (see Figure 6). 

**Figure 7:**
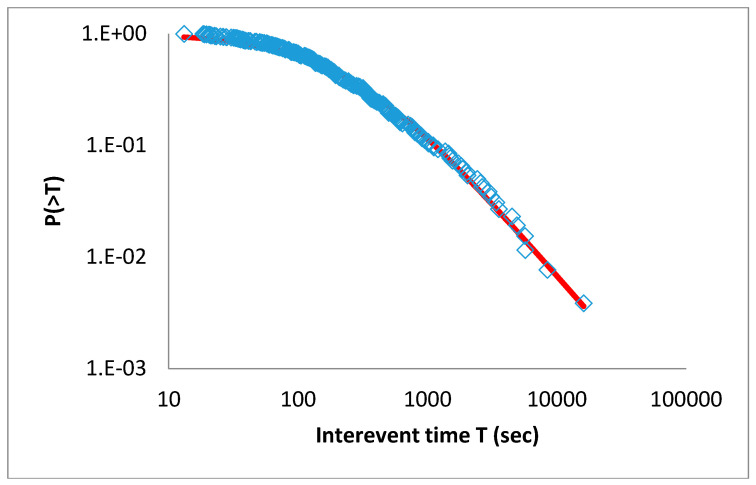
The cumulative distribution function of the interevent times *T* for the 2010 Madison Plateau earthquake swarm. The red line is the *q*-exponential fitting with *q_T_* = 1.745.

**Figure 8:**
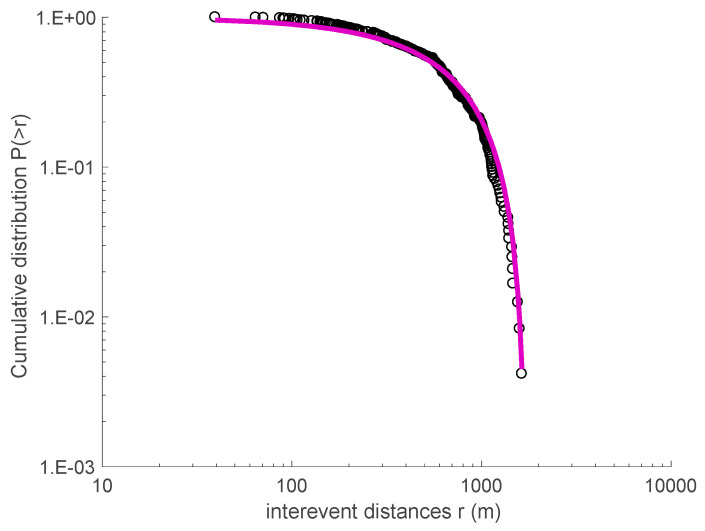
The cumulative distribution function for the interevent distances *r* of the 2010 Madison Plateau earthquake swarm. The black line is the *q*-exponential fitting with *q_r_* equal to 0.517.

A similar analysis for the Madison Plateau earthquake swarm (2010) leads to *q_T_* = 1.745 ± 0.065 and *T*_0_ = (1283 ± 48) s, and *q_r_* = 0.517 ± 0.036 and *r*_0_ = (1031 ± 21) m, respectively (see Figure 7 and Figure 8).

Our results indicate that the cumulative distribution functions of the inter-event times and distances between the successive earthquakes for the two swarms are well described by the *q*-exponential function (Equation (1)), with a *q* entropic parameter greater than one for the inter-event times and less than one for the inter-event distances, which is in agreement with [12,18,22,38,39,41]. 

### 3.2. The Magnitude-Frequency Distribution

The magnitude-frequency distribution of earthquakes can be described within the framework of NESP using the fragment-asperity model, as proposed in [32]. This model was firstly introduced in [43], and was modified in [44], estimating the fragment-size (*σ*) distribution as: p(σ)=[1−(1−q)(2−q)(σ−σq]11−q, where *σ_q_* is the *q*-expectation value of *σ*, as defined in [43,44]. The fragment-size distribution *p*(*σ*) expresses the probability of finding a fragment of surface *σ* and the proportionality of the released energy *E*, and the three dimensional fragments satisfies the expression: σ−σq=(EaE)23, where *σ* scales with *r*^2^ and *α_E_* is the proportionality constant between *E* and *r*^3^ (see [12,18,43,44] and references therein). Using the latter equation, the energy distribution function of earthquakes can be written as:

p(E)=1dEdσp[(EαE)23+σq]=dσdE[1−(1−q)(2−q)(EαE)23]11−q, with dσdE=23E−13αE23.

Combining the above relationships, the energy distribution function is obtained:

p(E)=C1E−13[1+C2E23]1qE−1 with C1=23αE23 and C2=−(1−qE)(2−qE)αE23. 

In the latter equation, the *q* index is replaced with $q_{E}$ to highlight that the Tsallis entropic index is related with the earthquake energy distribution. 

If *n*(*E*) corresponds to the number of earthquakes with energy *E* and *N* to the total number of earthquakes, considering that the probability of the energy is $p\left(E\right)=\frac{n\left(E\right)}{N}$, the cumulative number of earthquakes can be obtained as:

$$\frac{Ν\left(E>E_{th}\right)}{N}=\int_{E_{th}}^{\infty }p\left(E\right)dE,$$

where $\;N\left(E>E_{th}\right)$ represents the number of earthquakes with energy *E* greater that the threshold energy *E_th_*_._ Now the cumulative number of earthquakes becomes:

$$\;\frac{Ν\left(E>E_{th}\right)}{N}={\left[1-\left(\frac{1-q_{E}}{2-q_{E}}\right){\left(\frac{E}{\alpha_{E}}\right)}^{2/3}\right]}^{\frac{2-q_{E}}{1-q_{E}}}$$

According to [45], the earthquake magnitude M is related to the energy *E* as: $M=\frac{2}{3}logE$ and the previous equation leads into the expression [46,47]:

(2)  N(>M)N=[1−(1−qE2−qE)(10MαE23)]2−qE1−qE

In [32], the threshold magnitude M_0_ was introduced, which corresponds to the minimum earthquake magnitude of the catalog; the above Equation (2) is slightly changed into:

(3)  N(>M)N=[1−(1−qE2−qE)(10MαE23)1−(1−qE2−qE)(10M0αE23)]2−qE1−qE

**Figure 9:**
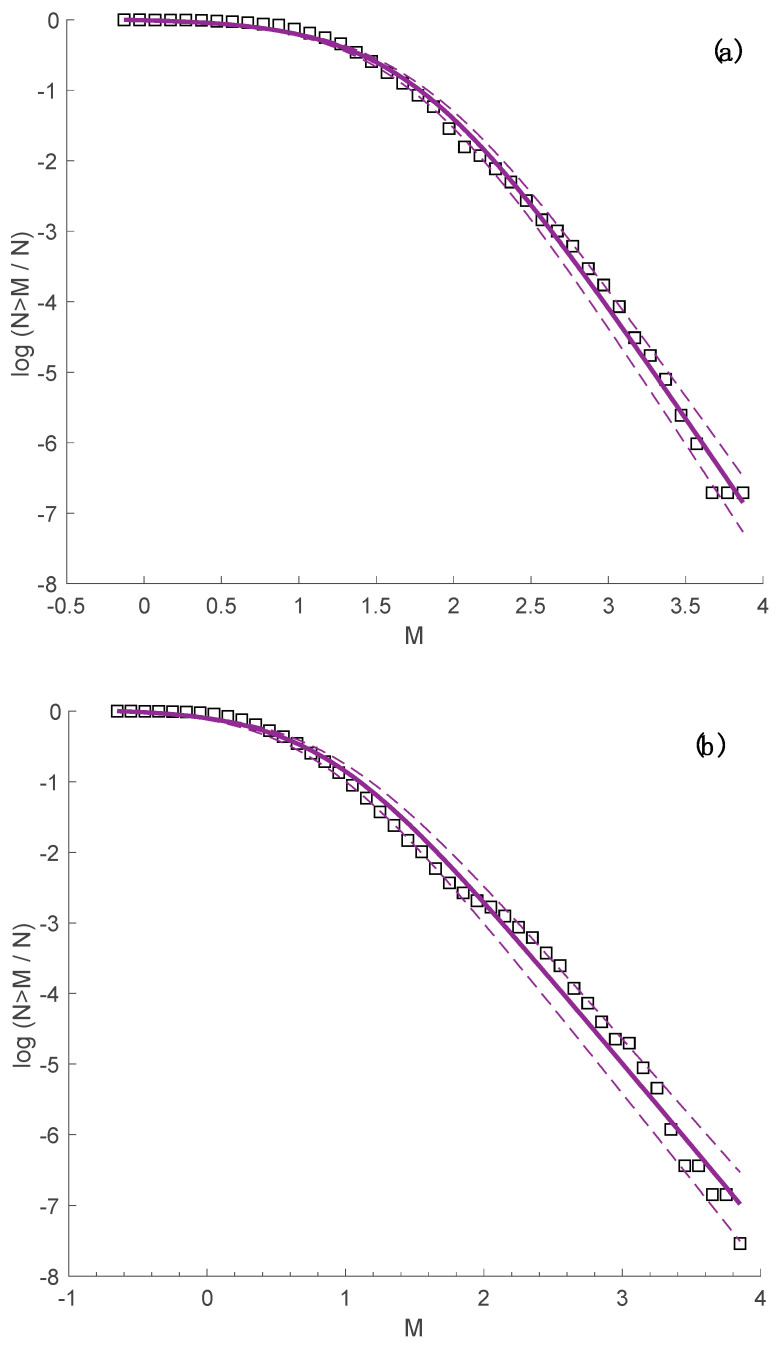
(**a**) The frequency-magnitude distribution of earthquakes for the 2008–2009 Yellowstone Lake swarm (squares) and the corresponding fir according to Equation (3) (solid line) for *q_E_* = 1.415. The two dashed lines represent the 95% confidence intervals. (**b**) The frequency-magnitude distribution of earthquakes for the 2010 Madison Plateau swarm (squares) and the corresponding fit according to Equation (3) (solid line) for *q_E_* = 1.496. The two dashed lines represent the 95% confidence intervals.

In References [32,43,44,46,47], it was shown that the fragment-asperity model describes the magnitude-frequency distribution of earthquakes, as proposed in the framework of NESP, introducing the entropic parameter *q_E_* that expresses the long-term interactions of the system under the constraints presented in [19]. The aforementioned model was applied to the two Yellowstone park earthquake swarms, and the results are presented in Figure 9a,b, for the 2008–2009 Yellowstone Lake swarm and the 2010 Madison Plateau earthquake swarm, respectively, while the fitting of Equation (3) to the observed data leads to *q_E_* = 1.415 ± 0.01 and *α_E_* = 257.9 ± 41.5 and *q_E_* = 1.496 ± 0.013 and *α_E_* = 18.8 ± 5.14, respectively. 

**Figure 10:**
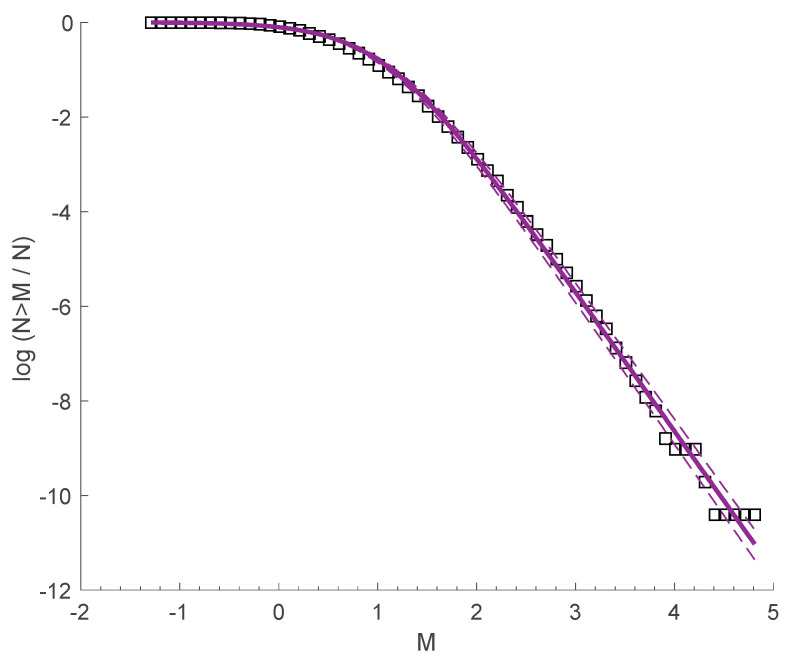
The frequency-magnitude distribution of earthquakes in the Yellowstone volcanic field from 1996 to 2016 (squares), along with the fitting based on Equation (3) (solid line) with *q_E_* = 1.44. The dashed lines represent the 95% confidence intervals.

From the analysis of the frequency-magnitude distribution of the two swarms, we can conclude that the fragment asperity model describes the seismic behavior and the energy release of the system very well. The distribution reflects a sub-extensive system, where long-range interactions are important, which is in agreement with previous results extracted from the analysis of several earthquakes catalogues [33,40,42] for different geotectonic environments. We note that the subadditivity of the two swarms agrees with the subadditivity of the frequency-magnitude distribution of earthquakes in the Yellowstone volcanic field from 1996 to 2016, where the values of *q_E_* = 1.44 ± 0.005 and *α_E_* = 27.65 ± 3.8 are found (see Figure 10).

**Figure 11:**
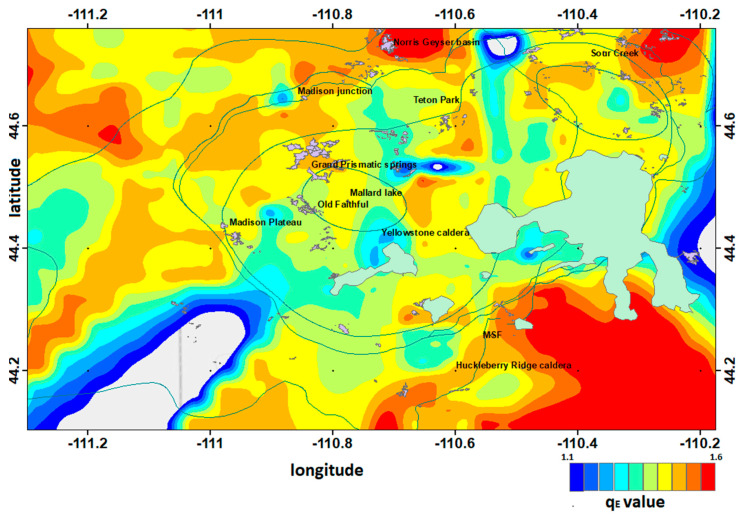
Spatial variation of the entropic index *q_E_* throughout the Yellowstone volcanic field.

The parameter *q_E_* describes the complexity of the tectonic system, which, in the case of Yellowstone seismicity, is affected not only by the tectonic history, but also—and in a crucial way—by the existing hydrothermal conditions, that can lead to high pore-pressures and pore-pressure diffusion associated with the fractures’ network. Motivated by the thermodynamic content of the entropic *q*-parameter, it is of interest to study the spatial distribution within the Yellowstone volcanic field. Using the frequency-magnitude distribution, the entropic parameter *q_E_* was spatially mapped throughout the Yellowstone volcanic area for the period of 1996 to 2016 in order to analyze its variability and to investigate its correlation with the innumerous hydrothermal processes that take place inside the Yellowstone area. Using Equation (3), *q_E_* value was estimated fitting the frequency-magnitude distribution inside a spatial squared window of 10 km length that was moving with an overlap of 2 km in order to achieve a smooth transition. Figure 11 presents the spatial variation of the entropic index *q_E_* throughout the Yellowstone volcanic field. An inspection of Figure 11 indicates that the entropic index *q_E_* throughout the volcanic field varies from 1.1 to 1.59, supporting subadditivity, while the smallest values are noticed in regions where several swarms have occurred in the past, such as in the Yellowstone lake (2008), the Madison plateau (2010), and along the NW edge of the park (1999). The greatest values are noticed east of the Sour Creek dome, an area that has experienced persistent seismic activity in the past and southwest of the Yellowstone caldera near Mt. Sheridan fault (MSF) until the northern edge of Teton fault (TF). We note that within the Yellowstone caldera, the values are smaller due to the existence of many hydrothermal features that increase heterogeneity and lead to high pore-pressure.

## 4. Concluding Remarks

In the present work, the framework of Non Extensive Statistical Physics (NESP) was used in order to analyze the spatial and temporal distribution of the inter-event distances and times among successive earthquakes that occurred during the two recent large swarms in the Yellowstone volcanic field. The calculation of the *q_T_* parameter resulted in values of 1.715 and 1.745 for the Yellowstone Lake and the Madison Plateau swarm, respectively. The cumulative distribution of the inter-event times presented power-law asymptotic behavior, leading to the conclusion that there are strong correlations among the events. At the cumulative distribution function for the inter-event distances, a cut-off appears, leading to *q_r_* values of 0.710 for the Yellowstone Lake swarm, and 0.517 for the Madison Plateau swarm. Our results, i.e., *q_T_* > 1 and *q_r_* < 1, are in agreement with those found in previous works, such as the Aigion aftershock sequence [35], the spatiotemporal properties of seismicity in California [38,40], and for global seismicity [42]. 

The concept of NESP describes well both the spatial and temporal behavior of the earthquakes among the 2008–2009 Yellowstone Lake swarm and the 2010 Madison Plateau earthquake swarm, supporting the idea that in a complex procedure, such as a seismic sequence, there is an obvious need to use complexity methodological tools in order to understand the scaling properties, considering that there are memory effects and the events of the sequence interact strongly with each other. The fragment-asperity model, as modified in [32], describes the energy distribution of the two swarms over a wide range of magnitudes; the entropic index *q_E_* is equal to 1.415 for the 2008–2009 Yellowstone Lake swarm and 1.496 for the 2010 Madison Plateau earthquake swarm. Furthermore, the subadditivity of the swarms is in agreement with the subadditivity concluded by analyzing the frequency-magnitude distribution of earthquakes in the Yellowstone volcanic field from 1996 to 2016, where *q_E_* = 1.44. 

The entropic index *q_E_* varies spatially throughout the Yellowstone area, with values in the range of 1.1 to 1.59 supporting subadditivity. The smallest values are noticed in regions where several swarms have occurred in the past, such as in the Yellowstone lake (2008), the Madison plateau (2010) and along the NW edge of the park (1999). The greatest values are noticed east of the Sour Creek dome, an area that has experienced persistent seismic activity in the past, and southwest of the Yellowstone caldera near Mt. Sheridan fault (MSF) until the northern edge of Teton fault (TF). We note that within the Yellowstone caldera, the values are smaller due to the existence of many hydrothermal features that increase heterogeneity and lead to high pore pressures. The latter supports the idea that the *q_E_*-parameter, due to its thermodynamic origin, could act as a fluid and temperature (i.e., heat flow) indicator, as proposed in [21]. 

The framework of NESP that was used in the present study describes well the spatiotemporal scaling properties of the two earthquake swarms, as well as the energy distribution, indicating the appropriateness of NESP in describing hydrothermal geosystems with hierarchical structure. The correlations among the earthquakes are strong; for this reason, the NESP approach seems to be an adequate tool for analyzing the spatiotemporal behavior of the two swarms and the hydrothermal features linked to seismicity in the Yellowstone tectonovolcanic environment.
